# Insights Into Glycobiology and the Protein-Glycan Interactome Using Glycan Microarray Technologies

**DOI:** 10.1016/j.mcpro.2024.100844

**Published:** 2024-09-21

**Authors:** Jamie Heimburg-Molinaro, Akul Y. Mehta, Catherine A. Tilton, Richard D. Cummings

**Affiliations:** Department of Surgery Beth Israel Deaconess Medical Center, National Center for Functional Glycomics (NCFG), Harvard Medical School, Boston, Massachusetts, USA

**Keywords:** array, glycan, glycan-binding protein, glycome, lectin, microarray

## Abstract

Glycans linked to proteins and lipids and also occurring in free forms have many functions, and these are partly elicited through specific interactions with glycan-binding proteins (GBPs). These include lectins, adhesins, toxins, hemagglutinins, growth factors, and enzymes, but antibodies can also bind glycans. While humans and other animals generate a vast repertoire of GBPs and different glycans in their glycomes, other organisms, including phage, microbes, protozoans, fungi, and plants also express glycans and GBPs, and these can also interact with their host glycans. This can be termed the protein-glycan interactome, and in nature is likely to be vast, but is so far very poorly described. Understanding the breadth of the protein-glycan interactome is also a key to unlocking our understanding of infectious diseases involving glycans, and immunology associated with antibodies binding to glycans. A key technological advance in this area has been the development of glycan microarrays. This is a display technology in which minute quantities of glycans are attached to the surfaces of slides or beads. This allows the arrayed glycans to be interrogated by GBPs and antibodies in a relatively high throughput approach, in which a protein may bind to one or more distinct glycans. Such binding can lead to novel insights and hypotheses regarding both the function of the GBP, the specificity of an antibody and the function of the glycan within the context of the protein-glycan interactome. This article focuses on the types of glycan microarray technologies currently available to study animal glycobiology and examples of breakthroughs aided by these technologies.

## Highlights

• Overview and studies of glycan-binding proteins and anti-glycan antibodies.
• Background of glycan microarrays and their utility and analysis.
• Insights into the protein-glycan interactome using glycan microarray technologies.
• Technological developments in glycan microarrays and bioinformatics.

## Graphical Abstract

**Figure:**
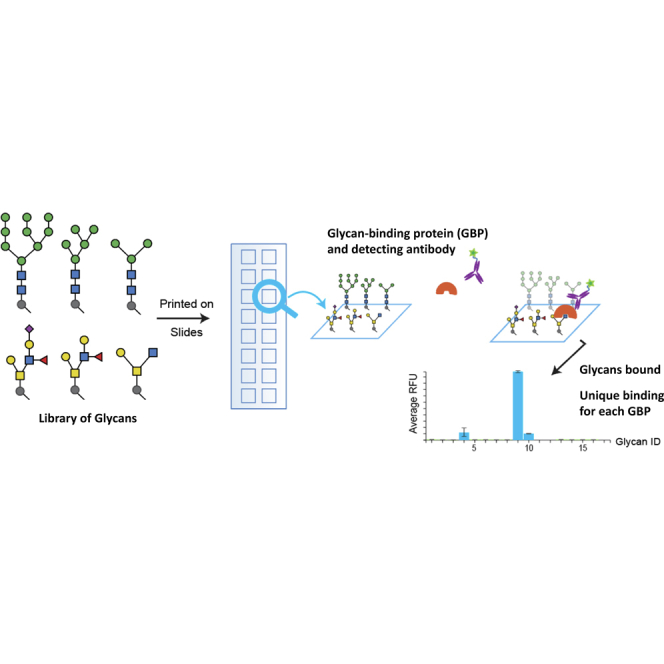

## In Brief

Here we discuss important advancements in glycan microarray technologies used to explore glycan recognition by glycan-binding proteins (GBPs) and antibodies. A significant outcome of such studies is the revelation that each GBP has a relatively unique specificity, suggesting that differential glycan recognition is associated with their unique functions. Glycan microarray technologies provide the potential to identify the Protein-Glycan Interactome within a host and for host-guest interactions and generate specific and testable hypotheses about glycan recognition and biological function.

## Background on Glycan-Binding Proteins

All organisms express glycans, which occur in a variety of glycomolecules, such as glycoproteins, glycolipids, and oligo/polysaccharides (1, 2, 3, 4, 5, 6, 7). Glycan expression in animal cells is essential for the survival of the organism due to their multiple roles in regulating adhesion, signaling, extracellular matrices, etc. In this regard, a key contribution of glycans to mammalian biology arises from their specific intermolecular interaction with proteins (6, 7, 8, 9). There are generally two types of proteins that interact with glycans—those that bind to glycans without altering them, and those that bind and alter their structure in some manner. The former are glycan-binding proteins (GBPs) (10) and include lectins (11, 12), agglutinins (13, 14), adhesins (15, 16), phage (17, 18), toxins (16, 19), and antibodies (20, 21), while the latter includes enzymes, for example, glycosyltransferases and glycohydrolases (22, 23). All of these proteins often have “canonical” glycan-binding motifs within their structures termed a carbohydrate-recognition domain (CRD) (24) or carbohydrate-binding module (CBM) (25), or the case of antibodies, an antigen-binding site that recognizes a glycoepitope or determinant (26, 27, 28). In the CAZy database, there are currently 101 such families of CBMs including GBPs and enzymes (29) (www.cazy.org/Carbohydrate-Binding-Modules.html). Some proteins have family members that are enzymes and others that have lost their activity yet retain glycan-binding activity, such as the multiple CBM groups of chitinases and chitin lectins (30). In addition, the HumanLectome (UniLectin portal https://unilectin.unige.ch/) provides information about a large number of human lectins and glycans to which they can bind (31). In consideration of lectins alone, ∼1.4 million different lectins have been predicted in nature (31), although many remain uncharacterized. For example, over 32,000 lectin sequences have been identified in fungi (32) and in the C-type lectin family, more than 1000 members are divided into 17 subgroups, with the caveat that the C-type lectin domain or C-type lectin domain in all those proteins may not always bind carbohydrates (33). In regard to microbes, the number of bacterial lectins in the human microbiome, many of which may bind human mucins and other glycomolecules, is enormous, and studies on them are still in the early stages (34).

In relation to this, the number of GBPs may be even larger, as many proteins that bind to carbohydrates do not have an easily identifiable CBM, such as proteins that bind glycosaminoglycans (35, 36). Such proteins are often not designated as lectins but as GAG-binding proteins. Hundreds of such GAG-binding proteins are known and include fibroblast growth factor-2 (FGF-2) (37) and antithrombin-III (AT-III) (38), and they can recognize GAG sequences with specificity and high affinity (39). Finally, there are those proteins that may be termed *non-canonical GBPs*; their structures and sequences may not indicate glycan-binding ability, yet such proteins may bind glycans under certain conditions. An example of this latter type is complement regulator factor H (FH), which binds to sialic acid but can also bind to heparin/heparan sulfate (40). Another example is the tandem LG domains in laminin, which have lectin activity and bind to the LARGE-glycan of α-dystroglycan (41). In addition, some proteins that lack a canonical CBM may bind carbohydrates with very low affinity, such as complement receptor 3 (CR3) (42) and α_M_β_2_ integrin (43). Thus, the actual diversity of GBPs in nature can only be partly predicted by protein structural motifs and will expand greatly using information from empirical studies to assign glycan-binding potential to a protein.

**Fig. 1:**
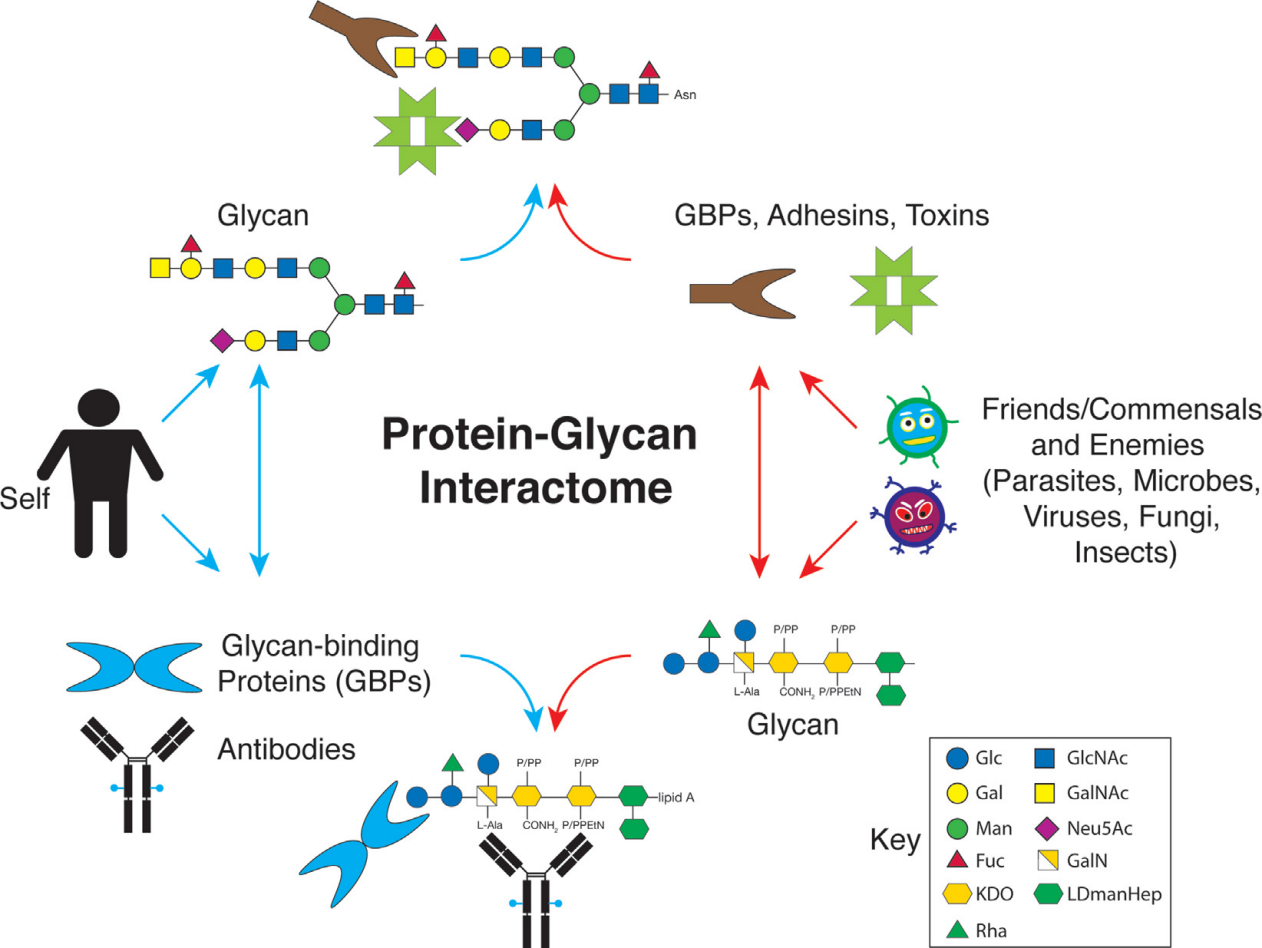
**Interconnections of the protein-glycan interactome**. Glycans expressed in mammalian systems can be targets for organisms and pathogenic organisms that express GBPs important in their invasion and other interactions with human cells; in turn, GBPs and antibodies made in mammals can target glycans in both animals and in other organisms and serve protective functions and also facilitate useful biological interactions.

Historically, this area of research has somewhat been divided into two broad approaches. One is focused on a GBP to define its specificity and biological functions, whereas the other is focused on a glycan to identify proteins that bind to it. In this context, one can envision a vast protein-glycan interactome in nature, in which all organisms in all domains of life - prokaryotes, archaea, and eukaryotes - express both glycans and GBPs that may interact within an organism or with another organism in some manner (1, 8) (Fig. 1).

Glycans that are recognized by GBPs are often termed ligands, counter-receptors, receptors, and binding partners. In general, any type of carbohydrate that binds a GBP, such as a monosaccharide or larger glycans, is termed a ligand. But whether these represent biologically important recognition or simply components of a natural glycan that can bind is unclear. The affinity differences between such glycans for their interactions may differ by orders of magnitude, and in some cases, the actual small molecule, e.g., disaccharide, may not exist in nature in that form or represent a very low-affinity binding partner. An example to consider is cholera toxin, which has pM affinity for the ganglioside GM1 (44), whereas cholera toxin can bind blood group antigens with mM affinity (45), although they have no direct structural similarity to GM1. Defining whether interactions are functionally important is a challenge. In addition, as glycans may be large and the binding sites of GBPs can be much smaller, GBPs may recognize a “motif” or “determinant,” that occurs as part of a glycan (26, 46), similar to how a protein epitope may be part of a large protein. Thus, the question arises, is the determinant alone sufficient for interaction, or does the glycan structure influence the interaction? Does a confluence or valency of such motifs confer additional affinity/avidity and specificity? These are difficult questions to definitively answer, as it might require vast knowledge of the expression of the glycan, determinants, biological expression of both glycans and GBPs, structural understanding of the GBP and its motif binding, and knowledge of the inter-organismal interactions, *i.e.* ecology, of the system. Unlike other macromolecules, glycans may be branched and have multiple internal and terminal motifs that influence their interactions with GBPs (47). In this regard, some GBPs probably function as pattern-recognition molecules involved in innate immunity that can bind with various affinities to determinants in multiple glyco molecules (48). The surface glycome or glycocalyx of cells is mysterious in many ways, in terms of associations of glycans, their own GBPs, and cis/trans interactions with free molecules or those in matrices, or on the same cells or other cells (49, 50, 51). Novel molecular engineering approaches are providing new insights into this complexity (52, 53, 54, 55, 56), for example, in regard to Wnt activity in binding heparan sulfate (57). Interestingly, the glycans expressed by an animal may serve as ligands for GBPs in another organism, as for viruses and microbes; such interactions might have negative or positive outcomes (58) (Fig. 1). This is seen to occur with pathological viruses that bind to human glycans, versus commensal bacteria that bind to human glycans (59).

Given its vast scope, it is not surprising that the diversity of the protein-glycan interactome is relatively unknown, as most glycans in nature are not yet described (1, 60, 61, 62), and most GBPs have not been studied in regard to their broad recognition of natural glycans and functions. Importantly, genetic studies are revealing the key biological roles of glycans in human biology, through the identification of heritable and *de novo* mutations in glycosylation pathways, e.g., congenital disorders of glycosylation (63, 64). However, there is often no specific connection between a glycan structure to its specific recognition in a biological system. While many glycans are essential to human and animal development, surprisingly only a few GBPs are currently known to be essential for normal development. Examples of essential GBPs include CLEC-2, which is expressed by platelets and is required for interacting with glycans on lymphatic endothelial cells to induce lymphangiogenesis (65, 66); calreticulin, a GBP in the ER that binds glucosylated N-glycans in glycoprotein quality control, which is required for normal cardiac development and regulation of intracellular calcium homeostasis (67); the cation-independent mannose 6-phosphate receptor, which is required for lysosomal hydrolase targeting to lysosomes (68), and whose loss results in fetal overgrowth and perinatal lethality (69); and GAG-binding proteins such as FGF-4 and hedgehog proteins that are critical for normal tissue development (70, 71).

However, while perhaps not required for development *per se*, many GBPs are essential for innate immunity and homeostasis, and while they might bind host glycans, their biological effects are often largely exerted through recognition of both host and xeno-glycans, i.e., those made by invading organisms or produced by non-human sources (72, 73). Such examples are DC-SIGN, a C-type lectin involved in pathogen recognition and antigen presentation in immune cells (74); the serum mannose-binding lectin, another C-type lectin in which mutations impair its function as an opsonin and its ability to induce complement by the lectin pathway (75); mannose-binding protein 1 (*LMAN1*) in which mutations lead to combined deficiency of coagulation factor V (FV) and FVIII (F5F8D) (76); and galectins that are important in immune regulation (77) and can also recognize microbial glycans and induce bacterial killing (78).

## Historical Perspectives on Identifying Glycan Recognition by Proteins

The need to characterize GBPs in terms of their specific glycan interactions was a driving force for the development of new technology platforms (79, 80, 81, 82). Such technologies address the question of how to explore the protein-glycan interactome, and how to identify specific glycans recognized by a GBP among other possible non-binding glycans? Historically, such questions have been very difficult to address, as they require access to glycans in nature, which are difficult to identify, sequence, and purify in significant quantities, and they require access to GBPs expressed either in their natural state, for example, membrane-bound or soluble or as recombinant proteins. A historical example of such difficulties was the identification of the specificity of antibodies to the blood group A antigen. Studies on this key biological area took many years to complete in the 1950s-1960s. It largely utilized glycoproteins and mucins purified from various tumors expressing blood group antigens, and their fractionation after partial acid hydrolysis to identify the smallest fragments exhibiting inhibitory activity toward antibodies that agglutinated cells of a certain blood type (83, 84). In related research areas on GBPs, researchers tested the ability of purified glycans to either inhibit or bind to a GBP as a way of defining its specificity, often using inhibition of agglutination with a small panel of inhibitory glycans or direct glycan binding to some small number of glycans (85). While these and related approaches provided insights into the specific binding of a GBP (86, 87, 88), few of them can be considered high-throughput and often lack the breadth of glycans needed for comparative testing.

Beginning in the 1980s, various solid-phase approaches became available to explore glycan recognition by proteins and offered the potential of higher throughput. These included lectin affinity chromatography, where mixtures of glycans were used and those that bound to a particular lectin could be affinity purified to define the specificity of the bound versus unbound glycans (89, 90, 91). Another compelling approach was thin layer chromatography of glycolipids and overlay of them for exploring their interactions with bacteria or GBPs (92, 93, 94). In addition, there were developments of a variety of ELISA-type or microtiter-plate-based arrays (95), and other approaches in which glycolipids (96) or neoglycoproteins (97) were immobilized and tested for binding by lectins or antibodies. All of these approaches have their advantages and limitations and many are still used in the field. Their limitations include the lack of glycan diversity to explore specificity, the relatively high amounts of glyco molecules needed for studies, the lack of community-wide access to the technologies, and the difficulty in adapting these approaches for generating high-throughput data. The microarray format was developed for nucleic acids (98), and the fundamentals of this technique were derived from gene arrays, in that the instruments and software developed for printing and image analysis could be used across disciplines. However, new developments and methods were needed to translate this method platform from genes to glycans, including surface chemistries for linking glycans. In addition, gene arrays usually include standards that can be applied with samples for quantification purposes, whereas glycans have to be quantitated separately before printing. This introduces a more difficult parameter for normalizing across laboratories but can be circumvented by including printing controls as well as assay standards as part of the experimentation and data analysis.

## Advantages of Glycan Microarrays

Beginning in the 2000s, this situation began to change with the introduction of glycan microarrays (79, 80, 81, 82) using either covalent or noncovalent linkage of glycans to surfaces, including the printing of glycans in multi-well platforms (99, 100, 101, 102, 103). In 2002, the Imperial College Carbohydrate Microarray facility began developing neoglycolipid-based oligosaccharide microarrays (79). Simultaneously, a major development was the establishment, through the support of the National Institute of General Medical Sciences (NIGMS), of a program termed the Consortium for Functional Glycomics (CFG). The CFG developed large libraries of hundreds of different glycans with functional linkers. These were first used as biotinylated forms in a streptavidin/ELISA-type array, and later the glycans were covalently printed to generate a large-scale glycan microarray, allowing high-quality control and more high-throughput analyses (80, 95). The CFG began collecting data from glycan microarray studies of GBPs of all types and this continues today through the National Center for Functional Glycomics (NCFG) supported by 10.13039/100000057NIGMS. Many thousands of glycan microarray studies have been performed and the data is available for analysis by scientists worldwide.

**Fig. 2:**
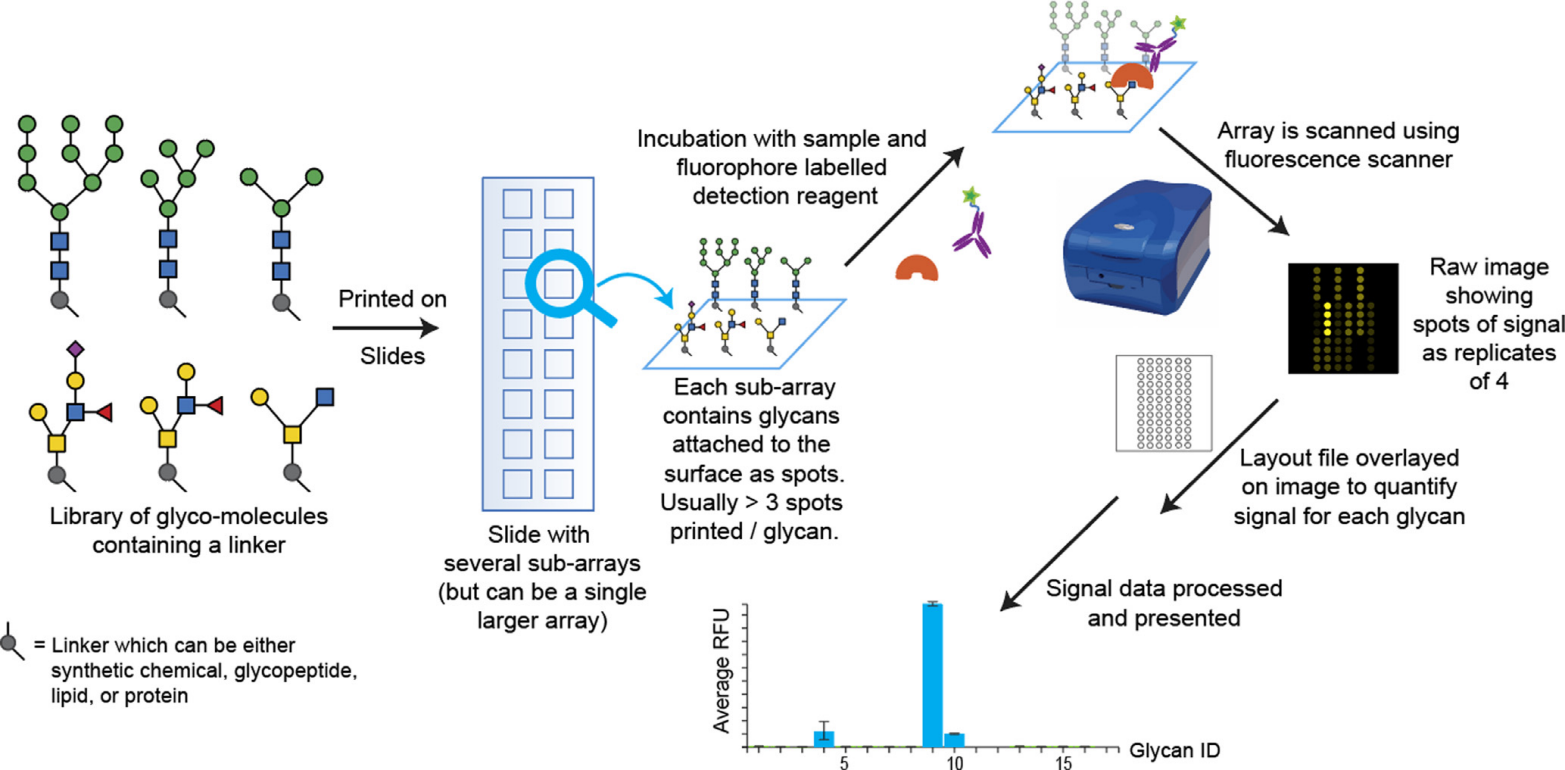
**Overview of the process of glycan microarrays, showing how libraries of glycans with linkers are printed on slides (usually glass with some functionalization or treatment).** These slides with the glycans attached are incubated with a sample (which is either fluorophore labeled or a secondary detection reagent with a fluorophore subsequently incubated). The sample may recognize specific glycan spots on the surface. The slide is then scanned using a fluorescence array scanner to generate a raw image, whose signal is quantified using a layout file with the scanner’s software and other data analysis software to produce the final signal data which is represented as the Average relative fluorescence units for each glycan.

The printed glycan microarray offers many advantages over other types of approaches, because all of the glycans being assessed are accurately printed on one slide (or subsection of a slide) in relatively equal amounts, and are assayed simultaneously in replicates by one sample preparation (Fig. 2) (80, 104). The assays are also performed on a relatively small scale which may require ∼10 to 25 μg of protein; can accommodate common buffer systems; and only ng of each printed glycan is present in a spot. When printing glycan microarrays, many slides can be printed in one batch and replicates for each glycan are printed and become inherent to the assay. Importantly, comparatively similar results were obtained when comparing samples on the printed glycan microarray of the CFG and its equivalent ELISA-based array using streptavidin-coated plates (95).

The breadth of glycans printed allows for a detailed study of the specificity of a GBP or antibody. Does the protein bind broadly to many types of glycans, or are a very restricted number of glycans bound? What related glycan structures are bound vs. unbound, and what are the relative binding values? Are high concentrations of the protein required, perhaps reflecting low affinity, or only very low concentrations, perhaps reflecting higher affinity? These types of analyses create a map of both the potential glycan specificity for each sample tested as well as a clue to its affinity or avidity. A range of concentrations can also be tested on the microarrays, with one single microarray surface generating data over several different concentrations. The presence of concentration-dependent binding provides further confidence that the protein-glycan interaction observed was reliable and helps to identify the “best” binders for a given sample and allows for ease of comparison with other samples. These best binders become *candidate ligands* that may then be further explored by orthogonal technologies, e.g., surface plasmon resonance (SPR), titration microcalorimetry, co-crystallization, etc. (88). Overall, the glycan microarrays are a robust platform for studying the protein-glycan interactome, and can be continually expanded as new glycans are generated or discovered, and are currently amenable to use with any sample that is soluble and can be detected fluorescently by direct or indirect methods.

## Different Types of Glycan Microarrays and Their Utility

### Classes of Glycans Covered

Current glycan microarrays cover a wide range of glycan types with defined glycan structures (20, 60, 105, 106, 107, 108). O-linked and N-linked-type glycans, glycosphingolipid glycans, GPI anchor glycans, glycosaminoglycan-type glycans, sialic acid-type glycans, milk oligosaccharides, and even glycopeptides and glycoproteins can be placed on microarrays, and the glycan sizes may range from monosaccharides to very large and complex structures, including tetraantennary N-glycans (47, 79, 109, 110, 111, 112, 113, 114, 115, 116, 117, 118, 119). Many of the glycan libraries involve glycans that are covalently printed on N-hydroxysuccinamide derived surfaces (80, 104, 120), but epoxide-activated glass slides are also used (121). There are also a number of different arrays containing glycopeptides, having at least the Ser, Thr, or Asn amino acid linkages intact (many with longer amino acid sequences), and a large variety of glycans represented on the glycopeptides, from single monosaccharides to large N-glycans (114, 122, 123, 124, 125). Glycoprotein arrays have also been generated and printed on nitrocellulose surfaces to better capture the glycoproteins (126, 127, 128). Similarly, neoglycoprotein arrays based on glycan linked to bovine serum albumin have also been created through deposition on nitrocellulose (129); in addition, neoglycolipid (NGL) arrays are also printed on nitrocellulose (106, 130). Microbial glycans or fragments/components of bacteria such as lipopolysaccharides and capsular polysaccharides have also been printed on N-hydroxysuccinamide slides, as well as intact bacteria printed on nitrocellulose (131, 132, 133, 134). Lipopolysaccharides and capsular polysaccharides are derived from bacteria with known glycan epitopes or a representative determinant known to be expressed and used to define the bacteria serologically. The materials in those cases are isolated from distinct bacterial species and printed to create microbial glycan microarrays (MGM) (133). Even whole bacteria have also been printed, representing the entire bacterial surface to study binding interactions of the whole organism (134).

A key limitation to the construction of a glycan microarray is the availability and cost of the defined glycans. Commercially available glycans that have a reducing terminus can be derivatized by various strategies using chemistries that are reactive with the reducing end, and then used to link the glycan to microarray surfaces; however, the cost of glycans and overall time for derivatization and subsequent purification can be significant. Alternatively, glycans can be synthesized through chemical and enzymatic methods in which the precursors already have linkers (79, 97, 99, 118, 128, 132, 135, 136, 137, 138, 139), but these are often costly, and also limited by current chemistry capabilities and available enzymes and acceptors. Naturally available and abundant glycans or glycopeptides of known structures can be isolated, derivatized, and printed, as has been done with N-glycans available from egg yolks and other natural products (114, 132, 140), and further modified enzymatically. This strategy can allow for the capture of relevant glycans from natural materials and can be greatly expanded upon, but the process is labor intensive.

To address some of these limitations, natural glycan array approaches have been developed, and these include *shotgun* glycan microarrays and *beam-search* arrays, in which undefined glycans are isolated from natural materials using various extraction methods and conjugated with a linker and printed without prior structural analysis (141, 142). In the case of shotgun microarrays, glycan material from a natural source retains many features of the native glycan (141, 142). Libraries of such endogenous glycans are maintained collectively and can be tested for glycan binding with biologically relevant materials. Examples of this platform utilized human and pig lung tissue, where glycans were isolated and printed as a shotgun microarray, and interrogated with influenza viruses, a known lung pathogen (8, 143). By studying the binding of the virus to the glycans and subsequent structural studies, new receptors, including sialylated and phosphorylated glycans, for the virus were identified in the human lung (see “Studies on Organism Interactions” section). Similarly, beam-search arrays, which are very sensitive compared to more standard methods, have been used to define the specificity of several anti-stem cell monoclonal antibodies and identify ligands for rotaviruses (144). Shotgun glycomics have also recently been employed with additional starting materials, including erythrocytes and materials from *Schistosoma mansoni*, *Caenorhabditis elegans*, and the whipworm *Trichuris suis* to interrogate binding interactions of GBPs and antibodies toward the native material (142, 145, 146, 147). The biggest hurdle to generating new shotgun glycan microarrays is the time and effort to derivatize, isolate, and separate the materials; however, once prepared they represent a unique and useful tool to study biological interactions and selectively target the interacting glycans of interest within the complex mixture.

### Glycan Array Availability

The CFG and MGM glycan microarrays publicly available through the NCFG are two types of microarrays that have been widely used by the research community for protein-glycan interactome studies. The CFG microarray represents one of the largest collections of chemoenzymatically synthesized glycans that are available on a single slide. Both the defined CFG and MGM microarrays are available at costs in the NCFG without the need for collaboration; many other more precious types of focused, defined, microarrays are also available upon request, such as the sialyl derivative microarray, oligomannose microarrays, glycopeptide microarrays, schistosome microarrays, as well as undefined shotgun microarrays such as for N-glycans from the human lung. The NCFG provides a full-service aspect, in terms of performing the binding assays, providing the analysis and help with interpretation and other support and resources.

Often laboratories generate exceptional glycan microarrays for their laboratory investigations, such as the Gildersleeve lab at the National Cancer Institute (NIH), and the Feizi Glycosciences Lab in London, and such arrays may be accessible through collaborations or service. The Gildersleeve platform contains many hundreds of neoglycoproteins, in which glycans are covalently linked to bovine serum albumin and they are then printed on nitrocellulose. One of their largest arrays combines glycans, glycopeptides, glycolipids, and glycoproteins on one array surface with a variety of linkers and carriers (118, 126, 148). The Feizi group uses the NGL base for the conjugation of glycans as pure compounds or as mixtures of amino lipids. The NGL technology uses a robotic printer to spot and immobilize the NGLs through hydrophobic lipid tails on nitrocellulose in a clustered, dense format (106). They have developed microarrays of a tremendous number of diverse glycan sequences including N- and O-glycans, glycolipids, glycosaminoglycans, and oligosaccharide components from bacterial, fungal, and plant polysaccharides (106, 149).

The Max Planck Society (MPS) glycan library was used to create a microbial glycan microarray unique from other arrays currently available (131). The MPS microbial glycan array consists of fully defined synthesized microbial glycans, in addition to mammalian glycans, and bacterial and parasitic fractionated glycan materials. It has proven to be useful in the study of human serum antibody repertoires, human lectin receptors, and bacterial lectins involved in virulence. The availability of generalized arrays with a large diversity of microbial glycans, and targeted arrays containing a single class of microbial glycans allows for a robust interrogation of glycan protein interactions surrounding glycoimmunology. This array may be available through collaborations or through commercial sources.

Above are examples of academic glycan microarrays available through centers and research laboratories. There are several commercial array vendors available, including but not limited to:

- Z Biotech (https://www.zbiotech.com/products/);
- GlycoNZ (http://www.glyconz.com/products/glycochip/);
- CD BioGlyco (https://www.bioglyco.com/glycobiology-microarray-platform.html);
- Creative Biolabs
  (https://www.creative-biolabs.com/glycoprotein/glycobiology-microarray.htm);
- RayBiotech (https://www.raybiotech.com/).

Commercial microarrays typically have a few hundred or so glycans per array and cover a range of glycan types, but suppliers can also provide focused, custom arrays. Compared to glycan microarray studies in collaboration with a center, such as the NCFG, users of commercial microarrays may not have full technical support available and specific controls, and this may complicate their use by novices in the field; in addition, some of the detailed information about array creation, e.g., linkers, densities, blocking reagents, and surface chemistry, may be proprietary and not available to the investigator. For such studies, an investigator typically needs to perform the assays in their laboratories, as well as have access to a slide scanner and software to analyze the data. Nevertheless, these commercial activities are very encouraging and demonstrate the wide appreciation and applicability of glycan microarray technologies.

### Modifications of Glycan Microarrays

An interesting variation on the use of a glycan microarray is to modify the existing glycans on the surface by enzymatic means for studying the activity of an enzyme or to create new glycan determinants on the array. Enzyme modification of glycan microarrays has been used for various applications in order to cleave glycans such as with the use of neuraminidase or PNGase F or A and analyze their activities and specificities, as well as to extend glycans such as with the use of T-synthase or ST6GalNAc1 glycosyltransferases (Table 1). The treatment of microarrays with enzymes can create a unique and new set of glycans compared to what was originally printed. This can result in an expanded array which is then able to be employed for additional assays, and compared to the unmodified array. Comparing binding on arrays with and without enzymatic treatments can allow for specific questions to be answered about binding profiles, such as the importance of sialic acid for binding. Furthermore, GBPs with enzymatic activity, such as viruses with sialidase activity, have been added to glycan microarrays in order to investigate which structures are altered and therefore provide insight into potential mechanisms involved in binding. The modification of microarrays using enzymatic treatments has proven to be useful and can expand the repertoire of information available from a single glycan microarray. Examples of array modifications are provided in Table 1.

**Table 1:** Examples of glycan microarray modifications

Publication title	Enzymes used	Reference
Novel lamprey antibody recognizes terminal sulfated galactose epitopes on mammalian glycoproteins	Neuraminidase PNGaseF	(127)
Parallel Glyco-SPOT Synthesis of Glycopeptide Libraries	ST6GalNAc1 T-synthase glycosyltransferase	(125)
Shotgun glycomics of pig lung identifies natural endogenous receptors for influenza viruses	Neuraminidase	(8)
A glycan array-based assay for the identification and characterization of plant glycosyltransferases	Plant glycosyltransferases	(218)

### Microarray Printing Technologies

Fabrication of glycan microarrays requires dispensing extremely small volumes of glycans as spots on a slide. There are various instruments available that are capable of performing this task, which can broadly be categorized into two dispensing technologies: (i) contact printers and (ii) non-contact printers.

Contact printers involve the dipping of pins (usually stainless steel) into a source material (usually in a microwell plate), followed by printing onto the substrate surface by making gentle contact between these pins and the surface. Given the nature of the deposition, care has to be taken with buffer selection and surface tension of the source material, so as to have proper adherence and ejection properties from the pins. A disadvantage of contact printers is the ambiguity of the exact volumes dispensed when contact is made. Also, due to continuous contact of the pins, microscopic damage over time can cause pins to wear. Examples of contact printers from the literature include NanoPrint from ArrayIt, SmartArrayer 136 from CapitalBio, SpotArray from PerkinElmer, Aushon 2470 arrayer from Quanterix. However, several of these are no longer available as users have shifted more toward non-contact printers.

Non-contact printers, as the name suggests, dispense the liquid to the substrate surface without making direct contact with the surface. The primary technology used for non-contact printing is piezo electric dispensing. In this, a piezo element (an element capable of contracting when voltage is applied), is placed around the fluidics path. The application of a voltage causes the contraction and expansion of the element, resulting in the expulsion of a nanodroplet. This piezo element can be housed either in nozzles or in print heads. This is the same technology used in inkjet printers and hence this is also called inkjet printing. Examples of non-contact printers in the literature include SciFlexArrayers from Scienion, Mercury from ArrayJet, M2-automation systems from axiVEND, and Nano-Plotter from GESIM.

Other considerations for printing revolve around maintaining the fidelity of the glycans themselves as well as the slide surface. If printing a large array with many glycan components effects the slide stability over time, then smaller numbers of glycans can be printed on one array. While this may increase the assay load, it will maintain the glycans and surface of the slide, resulting in better-quality assays and data.

### Microarray Scanner Technologies

Once an assay is performed, the array must be scanned using a scanner, to acquire an image for analysis. Usually, laser-based scanners are used for this purpose, such as GenePix scanners from Molecular Devices, InnoScan from Innopsys, and SureScan from Agilent. These scanners usually have lasers that can excite at wavelengths commonly used by detection fluorophores such as Alexa Fluor dyes (Alexa Fluor 488, Alexa Fluor 532, Alexa Fluor 635) and Cyanine dyes (Cy3 and Cy5). Signal intensities are controlled by the laser power and the sensitivity of the photomultiplier tube (PMT gain). Traditional glycan microarrays are typically scanned after being dried. However, a new method of wet scanning using a digital microscope imager could possibly be useful in also studying direct cell binding on glycan arrays (150).

Questions arise in the use of glycan microarrays to study GBP interactions concerning the binding affinity of the GBP. The typical concentrations for glycan microarray experiments use GBPs in the range of 5 to 200 μg/ml, so for a GBP at 50 μg/ml and a size of ∼50 kDa, that translates to a concentration of ∼1 μM. Thus, the binding of most proteins to the microarray is easily observable in this concentration range, but it is difficult to assign the dissociation constants (K_d_) or association constants (K_a_) to the interactions through the microarray approach. There are recent methods, for example, KdMining, which have been developed in this regard and appear very useful, where multiple concentrations of a protein are analyzed to provide a saturable binding isotherm (151). In any case, in consideration of the results from many thousands of analyses conducted at the NCFG, we have observed that GBP binding to typical printed glycan microarrays is detectable over a broad range of binding affinities, *e.g.*, K_d_'s of 10 μM to 10 nM; however, mM affinities are probably not observable As an example of the convergence of estimated affinities from glycan microarray analyses to more biophysical based observations, are studies on the cation-independent mannose-6-phosphate receptor (CI-MPR). The results indicated that its affinity for glycans containing mannose-6-phosphate, as measured by surface plasmon resonance, is in the range of K_d_ = 90 ± 6 nM (135). Correspondingly, when the His-tagged CI-MPR tested for binding at a concentration of 0.05 μg/ml (in the range of a few nM concentration) to such glycans on microarrays, binding was robustly and easily observed and different glycans exhibited differential binding on the microarrays that generally corresponded to different binding affinities observed by other methods (135). In addition, the issue of glycan density on a glycan microarray, while not well understood in a physico-chemical context, is clearly important (82). In that regard, different glycan densities are probably represented in different array formats, e.g., neoglycoprotein, neoglycolipid, printed glycan arrays, etc. Thus, while precise affinities are often not known for GBPs toward specific glycans on microarrays, the evidence indicates that if glycans are available on a microarray for binding by a GBP, both high- and low-affinity interactions are observable.

## Insights Into the Protein-Glycan Interactome Using Glycan Microarrays

### Lectin-Related Studies

Glycan microarrays are incredibly useful in providing insights into the specificity of many types of lectins. An early example of such insights is reflected in the study by Coombs *et al* (152), which utilized four mammalian C-type lectins (CTL) that were profiled in parallel on the CFG biotinylated glycan microarray. The rat-derived lectins, while known to bind galactose in general, showed distinct specificity. Rat macrophage galactose lectin, as well as human scavenger receptor CTL, bound mainly to Lewis x-type structures having a terminal Gal, as well as some GalNAc terminating structures. Conversely, rat hepatic lectin-1 and Kupffer cell receptors exhibited a much broader binding. While there may have been predictions and expectations of what each CTL would bind, the glycan microarray provided a robust and powerful tool for experimentally testing these observations, not easily accessible or comparable by any other means. Another example of the power of glycan microarrays regards the unusual specificity of LSECtin, a C-type lectin that uniquely recognizes terminal GlcNAcβ1-2Man residues on N-glycans (153).

While many GBPs bind to the CFG glycan microarray, there are some that do not, as they recognize types of glycans not available there. For example, intelectin-1, an intestinal mucosal protein (154, 155), exhibited no notable binding to the CFG glycan microarray, but it was later found that intelectin-1 bound well to specific glycans on the microbial glycan microarray (MGM) (133). The protein was observed to bind numerous glycan epitopes present on microbial surfaces, such as galactofuranose and KDO (156). The microarrays became a launch pad for detailed structural studies and bacterial interaction studies to characterize the function of intelectin-1.

Synthetic biology may be used in conjunction with glycan microarrays to explore glycan recognition versus biological activity (157, 158). An example of using glycan microarrays to engineer new glycan-binding reagents was the engineering of a lamprey-derived VLRB (O13), which recognizes the H-blood group antigen trisaccharide, but whose structure was altered in various ways to enhance its specificity for the H-trisaccharide (159). Similarly, a recent study by Wang *et al* (88) utilized a unique, defined microarray to test the power of rational design lectin-specificity for ligands. The binding specificity of E-selectin and Siglec-8 were identified by glycan microarrays, and computational engineering was used to make directed mutations to E-selectin with predicted outcomes in the binding specificity. These predictions were then experimentally tested on the glycan microarrays with wild-type and mutant proteins. Indeed, the microarrays were able to show the predicted change in specificity, but unexpectedly, a new binding partner was introduced in the mutant version. The glycans on the microarray, which contain sialylated and sulfated moieties, were instrumental in the outcomes of the study to understand engineering specificity and validate the predictions. Without the microarray results in tandem with the predictions, a critical ligand would have been missed. The microarray validation helps in turn to guide the algorithms used in the design.

Glycan microarrays are useful in defining the specificity of innate immune proteins, such as galectins, which has helped to understand their *in vivo* functions. To illustrate this, Stowell *et al* (160) used the CFG glycan microarray to decipher the separate binding domains of galectin-8 tandem repeat domains. By looking at the isolated domains, the contribution of each domain to binding was determined. The Gal-8N domain recognizes sulfated and sialylated glycans, determined by fluorescence anisotropy to have a 50 nM affinity for certain sialylated glycans (161), whereas the Gal-8C domain recognizes blood group antigens and poly-LacNAc glycans. This information provided an explanation for the immunological roles of galectin-8, and in the killing of microbes (134, 162), in the context of the biochemical assays done in parallel with the microarray assays. Similar differential domain activities and glycan specificities were mapped for galectin-9 using glycan microarray analyses (163).

### Studies on Organism Interactions

There is a long history of the use of glycan microarrays in virology studies, especially for defining the intricate binding to glycans by influenza viruses (113, 164, 165, 166, 167). Microarray studies can predict whether a virus has specificity for α-2,3 or α-2,6 linked sialic acids. However, they have proven to be much more informative in showing the importance of the underlying complex structures to which specific sialic acids are linked, and have helped to define their endogenous glycan receptors and better understand viral transmission.

For example, elegant studies by Stevens *et al* (168) used glycan microarray analyses to identify specific mutations in the 1918 H1N1 pandemic strains, that related to their specificities and preferences for α-2,6-linked sialic acid. This is important, as viruses infecting humans have a preference for this linkage (165), which is consistent with analyses of co-crystals of HA protein with glycans (169). Subsequent studies in recent years (164) analyzed receptor binding properties of an H1N1 pandemic virus derived from swine compared to seasonal strains (164). Swine are known to contain both α-2,3 and α-2,6 linked sialic acids in glycans of their respiratory tract and were thought to be a mixing vessel for human and avian-type viruses. The pandemic strain showed near exclusive binding to α-2,6 linked sialic acids, which is considered important for infection of human cells, and indicated that little to no adaptation appeared to occur in the binding component of HA for transmission from pig to human, and subsequent human to human spread. Interestingly, glycan microarrays also helped to identify influenza virus isolates that bound to glycans through their neuraminidase, as the hemagglutinin lost binding activity, as seen for isolates from 2006, 2010, and 2012 whose binding was inhibited by oseltamivir (170).

Recent studies on human and avian influenza viruses revealed that H3N2 viruses while maintaining their human-type specificity for α-2,6 linked sialic acids, have evolved a preference for glycans containing extended poly-LacNAc chains (166). Further studies demonstrated the unexpected ability of a single hemagglutinin trimer to increase its avidity by simultaneously binding to two branches of an extended N-glycan.

As pigs are a known reservoir for influenza, and due to the ease of obtaining material, pig lung was chosen as an ideal proof of concept for generating a shotgun glycan microarray (8). Total N-glycans were released from pig lung tissue, tagged, and separated to generate a glycan library, which was then printed to create a pig lung N-glycan shotgun microarray. Viruses were tested for binding, and each demonstrated differential specificity for the endogenous glycan ligands. This study opened the door to the idea of using the microarrays in an undefined fashion, to then follow up with structural studies on the glycans that proved to be the best receptors for viruses. This was advantageous over first identifying all of the glycan structures and then printing a *defined* microarray, as the structural analysis of all-natural glycans is incredibly labor intensive. This shotgun technology was extended to the human lung as a target tissue for influenza binding (143). This study also proved to be useful as an additional class of glycans, for example, phosphorylated glycans, emerged as a receptor for influenza viruses.

Overall, glycan microarrays have proven to be very useful in influenza studies, but have also shown utility in testing with coronaviruses including SARS-CoV-2 (171). Like the influenza virus, SARS-CoV-2 also bound to certain phosphorylated glycans (172). This may relate to the studies using glycosaminoglycan microarrays that show the virus can also bind chondroitin sulfate E (173) and other glycosaminoglycans (174), which is consistent with cell-based studies (175).

There have been several types of sialic acid glycan microarrays (176, 177). One of these was generated through unique one-pot chemistry (138), containing unique sialic acid modifications. Influenza and parainfluenza viruses were tested in this format, and evidence indicated that parainfluenza viruses could bind Neu5Ac and Neu5Gc derivatives. Influenza A was unable to bind Kdn and its derivatives, and interestingly bound equally well α-2,6 linked Neu5Ac9Lt (a lactoyl derivative) or the typical α-2,6 linked Neu5Ac. These studies provided novel insights into possible biologically relevant interactions.

### Applications Related to Antibodies and Immunity

The variety of glycan microarrays has proven to be useful platforms for immunological studies. In addition to immune-type lectins discussed elsewhere, antibodies to glycans are an ideal type of protein to test on glycan microarrays. Numerous studies have been performed to address questions of antibody responses to glycans, in both healthy settings and related to disease states. A large recent review covered this topic (20), and here are only some highlights of a few studies that showcase the findings of antibody studies on glycan microarrays.

It was incorrectly assumed earlier, based on responses of animals to immunization with pure glycans, that immune responses to glycan antigens are weak and typically represented by low-titer IgM class (178). Before the advent of glycan microarrays, it was not easily possible to test the breadth or affinity of serum immunoglobulins for glycan antigens. However, an early example of serum IgG binding to glycans on microarrays was the demonstration that patients with schistosomiasis expressed IgG and IgM capable of binding many unusual fucosylated glycans of the parasite *S. mansoni* (179). Initial studies with the CFG glycan microarray also demonstrated the binding of IgG and IgM in normal humans to specific glycans (80). Other studies using neoglycoprotein microarrays demonstrated that serum IgG binding to glycans was differentially affected by the density of conjugated glycans (180). The breadth of such binding of glycan antigens by human IgG was explored using intravenous immunoglobulin (IVIG) (181). IVIG is a collection of antibodies from thousands of healthy donors often used as a therapy for numerous diseases. Interestingly, about *half* of the glycans on the microarray were bound by IgG antibodies in the IVIG; those glycans generally did not represent human endogenous glycans. Removal of the IgG2 class of antibodies from the IVIG, thought to be the predominant anti-glycan antibody class, had only a modest effect on binding indicating that a broad class of IgGs bind glycans. Such unexpected results were entirely enabled by the breadth of glycans present in the microarray. A follow-up study (182) using a larger microarray and expanded sample set was able to draw broad conclusions on antibody binding, such as the identification of antibodies towards microbial antigens, as well as host glycans that function as attachment sites for pathogenic proteins, and tumor-associated antigens. These studies helped to show that the anti-glycan repertoire is an important feature of not only IVIG but of human plasma, and should be a consideration in therapeutics and vaccine design.

Glycan microarrays were unique in helping to identify individuals with primary antibody deficiencies (PAD), an affliction that leads to frequent infections and other sequelae in children (183). Strikingly, many patients were severely lacking in IgG antibodies to important glycans including microbial, some self-antigens, and tumor-associated glycans, although they maintained IgM responses. This defect in antibody repertoires pointed to an important feature of PAD and its specific IgG deficiency to carbohydrate, but not protein antigens, and could be a useful tool in monitoring the disease. A related study (184) also analyzed IgA binding patterns to microbial glycans, with interesting outcomes, including the binding of IgA1 and IgA2 to a diverse range of commensal bacteria. The mammalian-type and microbial arrays have proven to be extremely useful in understanding and comparing the antibody binding profiles to glycans and have put to rest the dated notion that antibodies to glycans are negligible, low-affinity, and of restricted specificity.

Other studies have looked at antibody responses in healthy individuals and different disease states. Many studies have examined anti-glycan antibodies in individuals with various cancers and metastatic tumors, and many unique antibody profiles have been identified associated with the disease (20). As this recent review gave detailed information about such cancer-associated antibodies and their potential as biomarkers that will not be further detailed here.

Anti-glycan antibodies have been useful in exploring many aspects of immunity and associations with disease and health beyond cancer. For example, an early study identified specific IgG and IgA antibodies to laminaribioside and chitobioside that may be useful as serologic markers associated with Crohn's disease (185). In terms of overall IgG and IgM antibodies to glycans in healthy individuals, they were explored using both animal and microbial types of microarrays (137). A profile of the IgG and IgM anti-carbohydrate antibody repertoires from 105 donors demonstrated that each individual had a unique IgG and IgM anti-carbohydrate antibody repertoire (ACAR), akin to a barcode of their immunity. Some antibody specificities were unexpected, and there were noted variations between individuals of different ages and ethnicities, suggesting that more detailed studies could be informative. Related studies using normal human serum have also identified many specific repertoires of anti-carbohydrate antibodies in healthy individuals (186, 187). Overall, the evidence strongly indicates that each individual’s ACAR provides a relatively unique pattern, suggesting that individualized information could be useful in precision medicine for predicting and monitoring health and resistance to disease.

Glycan microarrays have also been insightful in studying immune reactions to parasites. While parasites can be difficult to study due to their complex life cycle, creating microarrays of parasite-derived materials generated a new avenue for binding studies (124, 140, 146, 188, 189). Observations from these studies included the identification of immunogenic parasite glycans that were bound by IgG antibodies from infected individuals, such as FLDNF and core xylose/core fucose, raising the potential of novel glyco-based vaccines targeting these parasite-specific glycans that might be developed to combat diseases.

The abovementioned examples indicate a large number of key insights into the recognition of glycans and potential functions gained using glycan microarrays. As the number of insights is vast, only a few additional examples are presented in Table 2. These illustrate the use of printed glycan arrays and cell-based arrays to explore antibody binding to glycans, lectin specificities, and virus binding to glycans. As this review is not intended to compile all such studies using glycan microarrays, for which there are many hundreds, such citations highlight the invaluable nature and broad applicability of glycan arrays to explore biologically important glycan recognition.

**Table 2:** Examples of insights into glycobiology through glycan microarray technologies

Identification of antibodies to glycan antigens in patients with autoimmune disease, *e.g.*, Guillain-Barré syndrome, Type 1 diabetes (191, 219)
Specificity of influenza viruses for sialic acid and other potential ligands and neuraminidase activity (143, 166, 220, 221)
Antibodies to MUC1 glycopeptide epitopes in breast cancer (222)
Unusual specificity of mammalian lectins for microbial glycans, e.g., intelectin-1 (156)
Identification of 6′-sulfo-sLe^x^ as a ligand for human Siglec-8 (223)
Cell-based glycan array generated by modifying cell surfaces with enzymes to identify high affinity ligands for Siglec-15 (224)
Genetically engineered cell-based arrays to explore recognition of sulfated glycans in mucins (225)
Identification of serum antibodies to non-mammalian monosaccharide-containing glycans in microbes (131)
Use of sialic acid-type microarrays to identify unique specificities of sialic acid-recognizing reagents (226)
Identification of bivalve lectins that bind rhamnose and have antibacterial activity (227)
Shotgun glycomics of helminth parasite glycans to identify antigenic epitopes (146, 147)
Identification of 9-*O*-acetylated sialic acid as a ligand for SARS-CoV-2 Spike N-Terminal Domain (171)
Characterization of anti-hyaluronan antibodies in xenotransplantation (228)
Computationally-directed conversion of E-selectin to bind to sulfated ligands (88)
Discovery of novel human monoclonal IgG antibodies to glycan antigens (148)
Use of glycan microarrays to assess GBP binding affinities (151)
Exploration of ABO expression and antibodies to ABO in incompatible kidney transplantation (229)
Identifying the specificity of anti-fucose antibodies in sera of parasite infected individuals (188, 230)
Identification of children with primary antibody deficiencies lacking in IgG responses to microbial glycan antigens (183)
Human milk oligosaccharides are receptors for rotaviruses (231)
Identification of a universal sialoglycan-recognition of AB5 toxin B subunits (232)
Vast repertoire of IgG antibodies to glycan antigens in human serum (137, 181, 182)
Identification of ability of mannose-6-phosphate receptors to bind GlcNAc-6-mannose phosphodiesters (135)
Evidence that lipopolysaccharides of distinct bacterial pathogens independently bind directly to a range of host glycans through glycan-glycan interactions (233)
Development and characterization of specific monoclonal anti-glycan reagents using immunized lampreys (127, 159, 234)

### Breakthroughs in the Protein-Glycan Interactome Aided by Glycan Microarray Studies

Over the years, many thousands of experiments on glycan microarrays have been performed and data from these have been made available in many hundreds of publications and online literature. The microarrays produced by the CFG have been some of the most widely used arrays due to their large number (originally >600) of mammalian glycans offered per array. These microarrays have produced results for many distinct proteins, as illustrated in many of the above examples.

**Fig. 3:**
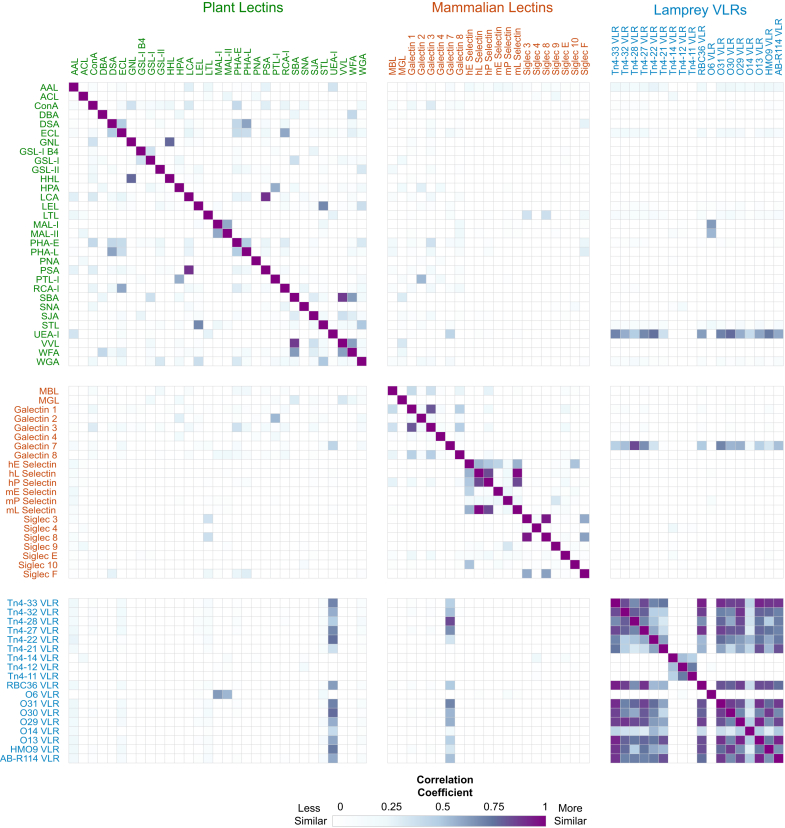
**Comparison of 70 glycan binding proteins.** Correlation map of 31 plant lectins, 21 mammalian lectins, and 18 lamprey VLRs, wherein the similarity of binding between each pair of proteins is compared and the Pearson correlation coefficient (r) is calculated and plotted as a heatmap where each tile represents the coefficient for that pair of proteins. The plot shows most proteins are quite different in their binding patterns as compared to others, but there are some proteins which have some correlation in binding.

The results raise the question – does each GBP and antibody tested on glycan microarrays exhibit unique specificity in glycan recognition? While reviewing all of the aggregate data produced using these CFG glycan microarrays is too vast to describe here, as an example of the diversity and impact of these results we analyzed 70 datasets. These include 31 plant lectins, 21 mammalian lectins, and 18 Lamprey variable lymphocyte receptors (VLRs) which were studied on the CFG microarray, in order to compare and show how glycan microarrays can provide valuable insights into finer glycan binding specificities (Fig. 3). We sorted the data to only retain those data points that were present in all datasets to avoid mismatches due to differences in microarray versions.

**Fig. 4:**
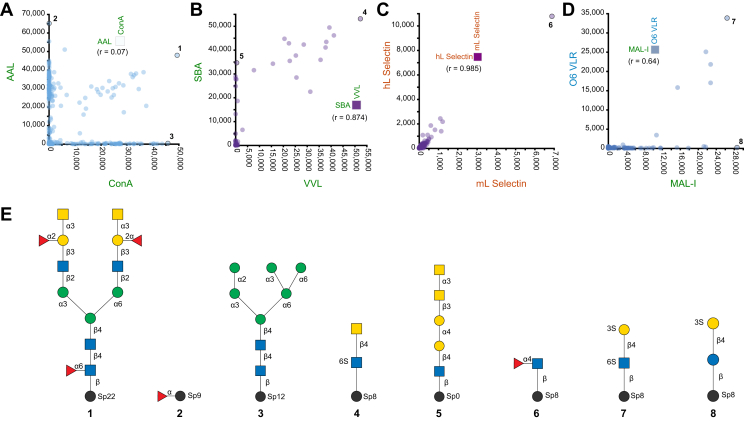
**One-to-one comparisons for selected proteins.***A*–*D*, scatterplots comparing pairs of GBPs to one another, where each circle represents a glycan on the microarray and the displacement in the x-axis and y-axis is determined by the binding signal intensity as measured in RFU. Some circles are outlined and a number is positioned adjacent to it, which can be matched to the structures shown in panel (*E*). *A*, comparison of AAL vs ConA as an example of proteins which bind very different motifs (r = 0.07). *B*, comparison of SBA and VVL which shows high correlation (r = 0.874), but there are some structures which selectively bind SBA and can be seen to be positioned close to the SBA axis (*e.g.*, structure 5). *C*, comparison of the same mammalian lectin from different species, in this case human vs mouse L-selectin, shows very similar binding patterns (r = 0.985). *D*, comparison of unique O6 VLR from Lamprey with the only co-relatable protein MAL-I with a weak correlation of r = 0.64. The O6 VLR selectively recognizes (3S)Gal attached to GlcNAc (structure 7) but does not recognize (3S)Gal attached to a Glc (structure 8). Data visualizations were made using GLAD (216), and glycan structures were drawn using GlycoGlyph (217).

Following that, we created a correlation map, where similar pairs of proteins would have a correlation coefficient of 1, while dissimilar proteins would have a correlation coefficient closer to 0. This map shows that most glycan binding proteins are very different in their binding patterns when compared to one another (Fig. 3). We looked more closely at individual protein comparisons (Fig. 4, *A*–*D*). For example, AAL and ConA show very diverse binding patterns and have an *r* value of 0.07. While both AAL and ConA do bind some structures that contain motifs recognized by both lectins (structure 1), AAL recognizes fucose binders (*e.g.* structure 2), while ConA binds to mannose as presented on N-glycans (structure 3) (Fig. 4*E*).

There are some pairs of proteins that are highly correlated and have somewhat similar binding patterns, yet there are clear differences between them. For example, SBA and VVL are both known to bind GalNAc, however, SBA recognizes GalNAc on additional structures that VVL does not bind. These distinctions in recognition between GBPs could be useful when they are used as detecting reagents, for example as stains on tissue sections, to hypothesize potential structures that may be present.

Mammalian lectins also show great diversity in their binding. However, the same protein between species does show similar binding. For example, human L-selectin (hL Selectin) vs. mouse L-selectin (mL Selectin) shows a very high correlation of 0.985. Classically, L-selectin is believed to bind to Sialyl Lewis X (sLeX) structures on proteins such as PSGL-1. However, on the arrays, the most prominent binders were glycans containing the Fucα1-4GlcNAcβ-R motif. Yet natural ligands of L-selectin do not have this structure (190). This highlights that glycan microarrays are tools to provide insights and hypotheses, and interpretations of biological significance should be made if contextually plausible and verifiable by other methods.

Lamprey VLRs are antibody molecules generated in sea lampreys that are unique in binding to carbohydrates. These VLRs could potentially be useful as reagents that identify unique glycan motifs. Lamprey VLRs can be quite similar to one another in identifying glycans, such as H-type 2 antigen structures. Glycan microarrays offer a way to identify unique VLRs that can differentiate between structures and therefore are exciting for further exploration. An example of this is the O6 VLR which quite uniquely bears a very low correlation to all other proteins in this analysis and recognizes terminal sulfated galactose (3S)Gal (127). The closest similarly binding protein is MAL-I in which both structures recognize terminal (3S)Gal. However, unlike MAL-I, O6 VLR seems to only recognize (3S)Gal attached in β1-4 linkage to a GlcNAc, as in (3S)Galβ1-4GlcNAc, but not to a Glc, as in (3S)Galβ1-4Glc. Other lamprey VLRs such as Tn4-11, Tn4-12, and Tn4-14 recognize Neu5Ac-containing structures but are unique compared to the entire dataset in correlation as well. Taken together, the lamprey VLRs can serve as a practical tool to map out the different glycan structures present in cells and tissues.

The correlation map (Fig. 3) brings together years of work over numerous laboratories, all asking the same overarching question: what are the relevant protein-glycan interactions? These types of data processing and analysis allow for comparisons to be made across different proteins, lectins, antibodies, and more, and as new data are obtained, it can be added to ongoing analyses. The most apparent observation is that each GBP and antibody has a relatively unique, identifiable profile of binding, even though some glycans may overlap between different samples.

The broader issue implies that for each glycan determinant in nature, for which the diversity is extraordinary (26), there may be a protein, for example, GBP, toxin, antibody, or adhesin, that recognizes it. It is also likely that vertebrate immune systems can generate antibodies to all types of non-self glycan structures. The observed diversity in glycan structures in nature and the diversity of such glycan recognition suggests a vastness of the protein-glycan interactome, which encompasses GBPs and glycans made by humans, animals, and microbes (Fig. 1), and the potential role of such interactions in competition and responses to pathogens or invaders (1).

## New Technological Developments in Glycan Microarrays and Analysis

### Luminex Glyco-Beads

Luminex is a bead-based assay performed using a similar method and concept as a glycan microarray, with the interactions occurring with both bead and sample in solution. When adapted to investigate protein-glycan interactions, distinct bead sets are coupled with glycans, which are then incubated with the sample of interest and fluorescent secondary detecting reagent. The assay becomes “multiplexed” by linking many different glycans to a distinct bead set, followed by mixing the bead sets together. This expands the glycans being interrogated, and these mixtures can then be assayed with different samples and appropriate secondary detection reagents (191, 192). This allows for a higher throughput nature of the assay. The creation of the glycan beads is the limiting step, due to the time required to generate them, as well as the necessity for ample amounts of each glycan. One major difference between the two techniques is the production of results. Classic microarrays result in a fluorescent image of bound samples, which must be aligned and analyzed by hand using analysis software. This is time-consuming and introduces some variability, including inter-reader variability. Luminex assays produce fluorescent signals instantly read by the machine, producing results within seconds. These features create opportunities for larger-scale analysis, where it is feasible to look at multiple samples with numerous secondaries each, due to the high throughput screening nature of this technology. This can be used in conjunction with glycan microarrays-a primary screen on a glycan microarray can narrow down the glycans bound, followed by Luminex assays to test a focused set of glycan beads with different samples and secondaries to make comparisons. An example of the utility of this method can be seen with serum sample studies, where the assays can be greatly expanded using Luminex beads, as well as using subtype antibodies (IgM, IgG, IgG1, IgG2, IgG3, IgG4, etc.). Recent Luminex-based platforms have been developed to explore serum antibodies to Group B *streptococcus* capsular polysaccharides coupled to Luminex beads, providing a rapid method to measure the specificity and titers of antibodies to vaccine candidates (193). Also, such approaches have identified clusters of anti-carbohydrate antibodies linked to progression to type 1 diabetes (191).

### DNA-Based Glycan Microarrays

Current slide-based microarrays consist of glycans printed in specific recorded locations, in which each location is used to identify a glycan. Next-Generation Glycan Microarrays consist of glycans conjugated to a unique DNA sequence which can be used to identify the glycan (194). Mixing of the oligonucleotide conjugated glycans with the GBP of interest, allows the bound glycans to be pulled down, and binding is then measured through PCR amplification. This assay requires smaller volumes of glycans and GBPs and can be used with a wide variety of GBPs such as cells, bacteria, and viruses, which can be difficult on classic glycan microarrays. Due to the reliance on PCR amplification, this method allows for a high throughput analysis of protein-glycan interactions in which specialized instrumentation is not required. A recent study also utilized Phage-displayed glycopeptides whose specific interactions with mannose-binding proteins could be identified by sequencing (195).

### Liquid Glycan Arrays

Liquid glycan arrays rely on the same principles as solid slide-based microarrays but are unique in that the effects of density on protein-glycan interactions can be investigated (196, 197, 198). Liquid glycan arrays consist of bacteriophages on which glycans are conjugated, along with a silent DNA barcode which is used to identify the corresponding glycans. Each phage-glycan conjugate can contain different densities of a single or multiple glycans. Because of this, dynamic competition can be observed as well as the measurement of affinity and avidity. Another advantage of this technique is that it is useful in studying protein-glycan interactions *in vitro*, like normal slide-based microarrays, as well as *in vivo*. Phage-glycan conjugates can be injected into animals and then the tissues of interest harvested to explore mechanisms of protein-glycan interactions.

### Bioinformatics

Over the years, several bioinformatics tools have been built around glycan microarrays and their data. We recently reviewed the bioinformatics tools in detail (199). Each tool has different functions and advantages over others, and is publicly accessible. Table 3 summarizes bioinformatics tools available, categorized into the following Types:

(i) Database: Tools for storage of datasets of experimental results for glycan arrays;
(ii) Data Management: Tools for managing glycan array experiment data and meta data as a performer of the assays;
(iii) Data Processing: Tools for processing the images of scans to help quantify the results of a microarray experiment;
(iv) Data Analysis: Tools that can be used to analyze and visualize processed data either manually or algorithmically to infer results from experimental data;
(v) GBP Binding Structure Analysis: Tools that provide 3D structural information for microarray results.

**Table 3:** Available bioinformatics tools

Tool	Type	Website	Reference
CFG Database	(i)	https://www.functionalglycomics.org	(235, 236)
Imperial College Microarray Data Portal	(i)	https://glycosciences.med.ic.ac.uk/data.html	
GlyGen Array Repository	(i)	https://glygen.ccrc.uga.edu/ggarray/	
CarbArrayART	(ii) and (iii)	https://glycosciences.med.ic.ac.uk/carbarrayart.html (http://carbarrayart.org/)	(237)
Glybrary	(ii) and (i)	https://glybrary.com/	
SignalFinder-Microarray	(iii)	https://haablab.vai.org/tools/	(238)
GLAD	(iv)	https://glycotoolkit.com/GLAD/	(216)
GlycoPattern	(iv)	Retired – but code for algorithm available at https://github.com/sagravat/glymmr	(239, 240)
MotifFinder	(iv)	https://haablab.vai.org/tools/	(241)
CarboGrove	(iv) and (i)^a^^,^^b^	https://carbogrove.org/	(242)
MCAW-DB	(iv) and (i)^a^^,^^b^	https://mcawdb.glycoinfo.org	(243)
CCARL	(iv)	https://github.com/andrewguy/CCARL	(244)
kdMining and kaPlotting	(iv)	https://data.mendeley.com/datasets/hrbtct6ryd/2	(151)
GlyMDB	(v)	http://www.glycanstructure.org/glymdb/	(245)
Gly-Spec (Grafting)	(v)	http://legacy.glycam.org/djdev/grafting/	(246)
Lectin Oracle	(iv)	https://github.com/BojarLab/LectinOracle	(247)

Abbreviation: CFG, Consortium for Functional Glycomics.

a In transition to a new platform during time of writing.

b Contains pre-existing datasets reanalyzed using algorithms (may allow upload of new datasets for analysis).

### Reusable Glycan Microarrays

Since the development of glycan microarrays, each array experiment is typically limited to assaying a single sample per slide. After binding of proteins, the slides are dried and images are acquired in a scanner; the drying of the slide results in irreversible binding of the GBP to the slide surface, by mechanisms not fully understood. Early attempts at removing bound materials resulted in decreased signals in subsequent assays and high background. Microwave-assisted wet-erase is a new method in which the slides are not dried, and after imaging in a wet condition, the GBPs can be removed from the slide while keeping the integrity of the printed glycans, allowing the microarray to be reused with high accuracy (150). Implementation of the microwave-assisted wet-erase method allows for the value of a glycan microarray to increase and extends the use of the precious glycans printed on the array. Extending the usage of a single array allows for access to information on protein-glycan interactions for at least 15 times more samples, which is significant given the limited access to and number of available arrays. Reliability and consistency within a sample set also increase when using the same slide by eliminating any printing differences between each array. This method will likely be broadened to other types of arrays and samples, and be coupled with enzyme treatments.

### Novel Developments and Alternative Platforms

Other developments, such as a label-free detection protocol where unlabeled or underivatized proteins can be bound to the glycan array and labeled after binding, are in development and will allow additional questions to be answered on the glycan arrays. For example, a label-free approach has been developed using surface-enhanced Raman spectroscopy to examine the binding of many different glycans to a variety of galectins and influenza virus hemagglutinins (200). Another recent study used a glycan-based receptor analogue microarrays on the Arrayed Imaging Reflectometry or AIR platform. These arrays demonstrate label-free, multiplex detection using biotinylated glycans allowing identification of differences in binding between human and avian influenza viruses (201). In addition, many studies are revealing the potential of using mass spectrometry-based approaches to determine the specificity and dissociation constants of GBPs toward glycan ligands (202, 203). The expansion of label-free technologies should speed up the development of new insights into the protein-glycan interactome without the burdensome preparation of recombinant GBPs with epitope tags and their detection methods. Such new technologies will also enable the possibility of the arrays as a discovery platform, by allowing unknown and unlabeled materials to bind to the array, followed by specific removal of the bound material and identification by proteomics methods. Features including the wet scanner printing larger spot sizes, and coupling the glycan microarray technology to other robust methods such as proteomics are key to bringing this idea of discovering new GBPs to fruition.

Alternative types of glycan microarray platforms are already in practice to explore glycan interactions from a different perspective. This includes lectin microarrays (204, 205, 206), which represent a reversal of the interaction by printing lectins to look at glyco-materials to which they bind. Such studies that exploit defined specificities of lectins are typically used to reveal features of natural-derived glycans, but such approaches could probably be adapted to identify novel glycans. These have a broader characterization outcome due to the nature of lectins, but novel observations can be made with these types of arrays, such as identifying new bacterial glycans able to bind to mammalian C-type lectins (205). There are also microarrays where antibodies are printed, which by nature is a more specific interaction that can be identified (207). In that type of approach, a specific anti-Tn antigen antibody was used to capture Tn-positive materials, to generate a cancer-detection platform. There is potential for expansion to other specific antibodies to be used to widen the screening platform to other types of glycans associated with cancers and other diseases. A variation of this theme is the development of an antibody-array to capture cells, and then using MALDI-IMS to analyze cellular N-glycosylation of bound materials (208). While not a direct protein-glycan interaction, the technology could be adapted to use anti-glycan antibodies as capture reagents. NMR could also be a useful technique in studying glycan-protein interactions in a low throughput manner but with superior structural details (209).

## Summary and Future Directions for Studying the Protein-Glycan Interactome

Here we have surveyed aspects of the important roles of glycan microarray technologies in exploring glycan-binding specificity of a variety of GBPs and antibodies. The field is expanding rapidly and this review has provided only highlights of important past and present developments. Clearly, the protein-glycan interactome is vast and significant, but it is not well studied or understood, and glycan microarrays in various formats present a valuable approach with the potential to be relatively high-throughout. So far, a significant outcome of glycan microarray technologies is the revelation that each GBP has a relatively unique specificity, suggesting a differential glycan recognition in their functions. The expansion of the glycan microarray platforms, in connection with the bioinformatics tools already in use and in development, will help to understand the biological significance of these interactions and their interconnectivity in a broader view.

While successful in a revolutionary way for expanding functional glycomics, glycan microarrays still have limitations. These are primarily in the (i) diversity of glycans, (ii) breadth of their structures and specific determinants, (iii) types of linkers and presentations, (iv) detection methods used, (v) need to develop reusable microarrays, (vi) costs and access to glycan microarray technologies, (vii) need to include presentation of glycans that may more closely mimic natural presentation on cell surfaces and the extracellular matrix and many others. The potential of variations on shotgun glycomics, which combines glycomic analyses with natural glycan-recognition could pay dividends in this direction. Glycan microarrays currently are often used to explore specificities of known GBPs, but they may also be useful in identifying novel GBPs through combinations of mass spectrometry and anti-protein mAbs and aptamers, which represent a new category of artificial molecules that can bind to glycans and other glycoforms (210, 211, 212). It is intriguing to speculate that because many glycans are expressed in very high densities on cells and in glyco molecules, affinities/avidities of GBPs need not be high in regard to participating in key glycan recognition and functions, whereas existing glycan microarray technologies require relatively high affinity. Neoglycoprotein and other presentation platforms might be important in providing spatial density of glycans mimicking that in nature, and future work that delves deeper into the density question related to the glycan microarray density across platforms as compared to the biological setting is warranted. Novel and low-affinity protein-protein interactions are being mapped using innovative computational methods and mass spectrometry-based approaches (213).

Interestingly, it is commonly accepted that the vast majority of proteins in the proteome bind to other proteins (214). This raises the question as to whether a large number of our proteins can also bind to glycans, perhaps with low affinity, and perhaps at sites independently of protein binding regions. Such a feature occurs in galectins, where in their intracellular capacity they bind protein partners, and in their extracellular capacity they bind glycan ligands (78). Similar multi-domain functions exist in the mannose-6-phosphate receptor, which can interact with other ligands in other domains, such as insulin-like growth factor-II (IGF-II) and many others (215). In that regard, it is also intriguing to consider linking aptamer-based proteomic technologies to identify not just the proteome but those GBPs within the proteome, perhaps not yet known to bind glycans. Nevertheless, in a relatively short time of a few decades, glycan microarray technologies provide the potential to identify the protein-glycan interactome and generate specific and testable hypotheses about glycan recognition. One can hardly imagine a future in glycobiology in which glycan microarrays and variations on such approaches do not play a crucial role in discovery.

## Data availability

All representative data are contained within the article or at the NCFG website.

## Conflicts of interest

The authors declare that they have no conflicts of interest with the contents of this article.
